# Programmed Death-Ligand 2 Deficiency Exacerbates Experimental Autoimmune Myocarditis in Mice

**DOI:** 10.3390/ijms22031426

**Published:** 2021-01-31

**Authors:** Siqi Li, Kazuko Tajiri, Nobuyuki Murakoshi, DongZhu Xu, Saori Yonebayashi, Yuta Okabe, Zixun Yuan, Duo Feng, Keiko Inoue, Kazuhiro Aonuma, Yuzuno Shimoda, Zoughu Song, Haruka Mori, Honglan Huang, Kazutaka Aonuma, Masaki Ieda

**Affiliations:** 1 Department of Cardiology, Faculty of Medicine, University of Tsukuba, Tsukuba 305-8575, Japan; l.siqi@outlook.com (S.L.); n.murakoshi@md.tsukuba.ac.jp (N.M.); xu_dongzhu@md.tsukuba.ac.jp (D.X.); syonebayashi789@gmail.com (S.Y.); yokabe0211@gmail.com (Y.O.); s1930477@s.tsukuba.ac.jp (Z.Y.); fengduoryu@outlook.com (D.F.); s2030351@s.tsukuba.ac.jp (K.I.); kazuaonuma.x19@gmail.com (K.A.); s2021402@s.tsukuba.ac.jp (Y.S.); s2030407@s.tsukuba.ac.jp (Z.S.); s1711822@s.tsukuba.ac.jp (H.M.); kaonuma@md.tsukuba.ac.jp (K.A.); mieda@md.tsukuba.ac.jp (M.I.); 2 Department of Pathogenobioligy, College of Basic Medical Sciences, Jilin University, Changchun 130012, China; hhl@jlu.edu.cn

**Keywords:** cardio-oncology, EAM, myocarditis, immune checkpoint, PD-L2, autoimmunity

## Abstract

Programmed death ligand 2 (PD-L2) is the second ligand of programmed death 1 (PD-1) protein. In autoimmune myocarditis, the protective roles of PD-1 and its first ligand programmed death ligand 1 (PD-L1) have been well documented; however, the role of PD-L2 remains unknown. In this study, we report that PD-L2 deficiency exacerbates myocardial inflammation in mice with experimental autoimmune myocarditis (EAM). EAM was established in wild-type (WT) and PD-L2-deficient mice by immunization with murine cardiac myosin peptide. We found that PD-L2-deficient mice had more serious inflammatory infiltration in the heart and a significantly higher myocarditis severity score than WT mice. PD-L2-deficient dendritic cells (DCs) enhanced CD4^+^ T cell proliferation in the presence of T cell receptor and CD28 signaling. These data suggest that PD-L2 on DCs protects against autoreactive CD4^+^ T cell expansion and severe inflammation in mice with EAM.

## 1. Introduction

The programmed cell death protein 1 (PD-1) pathway has been shown to be an attractive target for cancer immunotherapy. Immune checkpoint inhibitors targeting PD-1 or one of its ligands such as the programmed cell death ligand 1 (PD-L1) improves the prognosis of various malignancies [1,2]. However, the clinical benefit provided by these treatments can be accompanied by a unique and distinct spectrum of immune-related adverse events. Among them, myocarditis is a relatively rare, but potentially life-threatening cardiovascular side effect; therefore, it has become an emergent problem for clinicians in their daily routines [2,3]. Given these clinical aspects, a better understanding of how disruption of this pathway influences the immune system in the heart is needed.

The protective roles of PD-1 and PD-L1 in cardiac immune homeostasis have been well established [4,5,6,7,8]. PD-1^−/−^ mice on a BALB/c background developed autoimmune dilated cardiomyopathy, with the production of autoantibodies against cardiac troponin I [4,5]. PD-1 or PD-L1 deficiency in the autoimmune-prone MRL mice induced lymphocytic myocarditis with massive infiltration of CD4^+^ and CD8^+^ T cells [6,7]. Moreover, administration of anti-PD-1 antibodies exacerbated cardiac myosin peptide-induced experimental autoimmune myocarditis (EAM) [9]. However, the role of PD-L2, the second ligand of PD-1, in cardiac autoimmunity remains unknown.

In this study, we investigated the effect of PD-L2 deficiency on the development of myocarditis in the EAM model. We found that PD-L2 deficiency caused severe inflammatory infiltration in the heart. PD-L2-deficient dendritic cells (DCs) enhanced CD4^+^ T cell proliferation in the presence of the T cell receptor (TCR) and CD28 signaling, suggesting that PD-L2 on DCs protects against cardiac antigen-reactive CD4^+^ T cell expansion and severe inflammation in mice with EAM.

## 2. Results

### 2.1. Expression Levels of PD-1 and PD-Ls in the EAM Hearts

**Figure 1:**
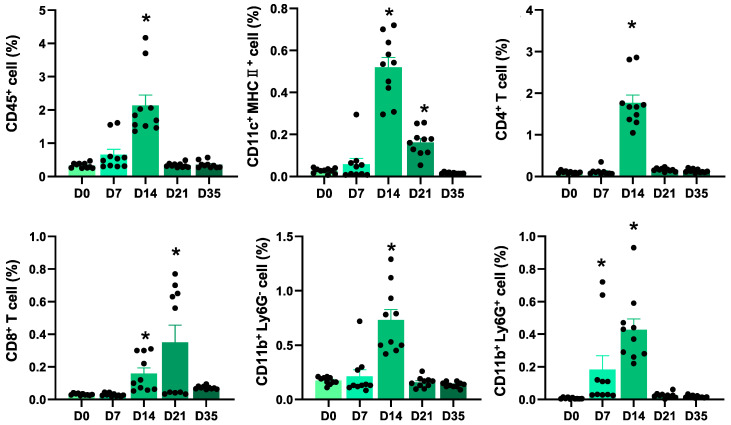
Dynamics of inflammatory cells in cardiac myosin peptide-induced autoimmune myocarditis. Quantification of inflammatory cells in the hearts obtained from mice with experimental autoimmune myocarditis, represented as a percentage of live cells at the indicated time points (n = 10 at each time point). The values are expressed as the mean ± standard error of the mean. * *p* < 0.05 vs. day 0.

We first examined the dynamics of inflammatory cells in EAM hearts. The heart inflammation peaked at 14 days after the first immunization with cardiac myosin peptide as evidenced by an increase in the number of infiltrating CD45^+^ leukocytes, CD11c^+^ dendritic cells (DCs), CD4^+^ T cells, CD11b^+^Ly6G^−^ monocytes/macrophages, and CD11b^+^ Ly6G^+^ neutrophils (Figure 1). The number of infiltrating CD8^+^ T cells peaked on day 21 (Figure 1).

**Figure 2:**
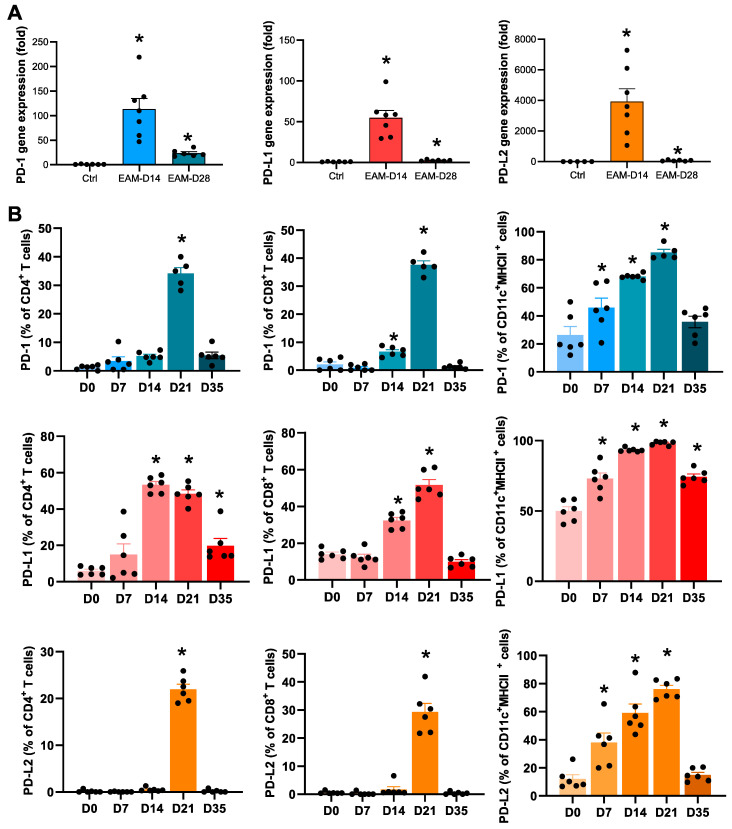
Expression levels of programmed cell death protein 1 (PD-1) and programmed cell death ligands (PD-Ls) in the experimental autoimmune myocarditis (EAM) hearts. (**A**) mRNA expression of PD-1, PD-L1, and PD-L2 in the hearts obtained from mice with EAM (days 14 and 28) and control mice (n = 6–7). (**B**) Flow cytometric analysis of the expression of PD-1, PD-L1, or PD-L2 on infiltrating CD4^+^ T cells, CD8^+^ T cells, and CD11c^+^MHC II^+^ dendritic cells in the hearts obtained from mice with EAM on days 0, 7, 14, 21, and 35 after first immunization with cardiac myosin peptide (n = 6 at each time point). Results are presented as the mean ± standard error of the mean. * *p* < 0.05 vs. day 0.

Next, we examined the expression levels of PD-1, PD-L1, and PD-L2 in the hearts of mice with EAM. The mRNA expression levels of PD-1, PD-L1, and PD-L2 were markedly upregulated on day 14 in the EAM hearts (Figure 2A). Particularly, PD-L2 gene expression increased approximately 4000-fold compared to the control (Figure 2A). Flow cytometric analyses revealed that the expression of PD-1, PD-L1, and PD-L2 on CD4^+^ T cells, CD8^+^ T cells, and CD11c^+^ DCs peaked around 14–21 days after the first immunization (Figure 2B). Thus, the expression levels of PD-L2, as well as PD-1 and PD-L1, were upregulated in the heart during cardiac inflammation.

### 2.2. PD-L2 Deficiency Exacerbates Cardiac Inflammation in EAM

We then examined the functional significance of PD-L2 in EAM. BALB/c mice are highly susceptible to myocarditis [10], and PD-1-deficient BALB/c mice spontaneously develop autoimmune dilated cardiomyopathy with the production of autoantibodies against cardiac troponin I [4,5]. On the other hand, C57BL/6J mice are resistant to myocarditis [11], and PD-1^−/−^, PD-L1^−/−^, or PD-L2^−/−^ mice on the C57BL/6J background do not develop spontaneous myocarditis and dilated cardiomyopathy [12]. Therefore, to investigate the effect of PD-L2 on the development of cardiac myosin peptide-induced myocarditis, we used F1 hybrids of PD-L2^−/−^C57BL/6J mice and BALB/c mice (PD-L2^+/−^).

**Figure 3:**
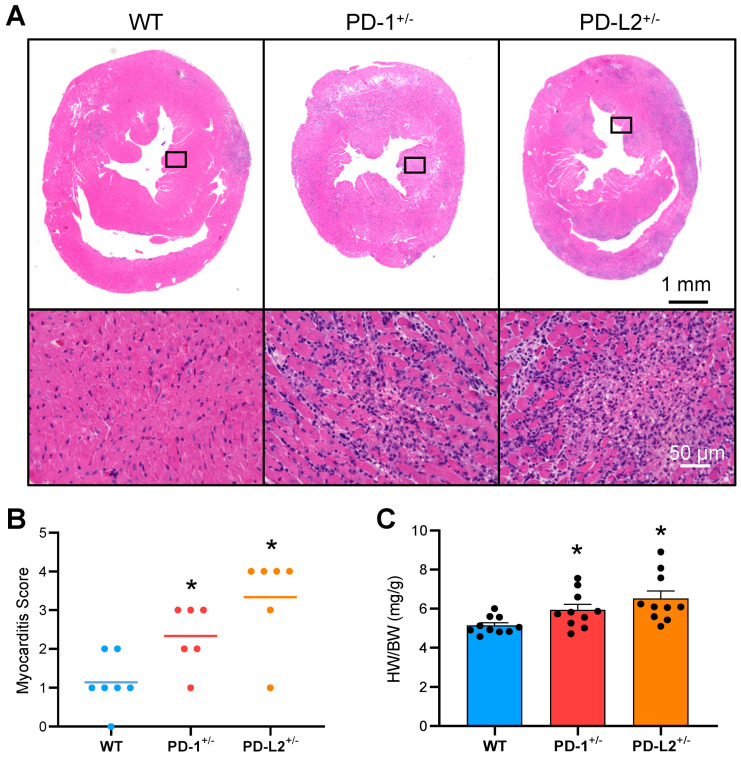
PD-L2 deficiency exacerbates myocardial inflammation in experimental autoimmune myocarditis. F1 hybrids of PD-1^−/−^, PD-L2^−/−^, and wild-type (WT) mice on the C57BL/6J background and BALB/c mice (PD-1^+/−^, PD-L2^+/−^, and WT, respectively) were immunized twice, on days 0 and 7, with cardiac myosin epitope peptide. (**A**) Representative H&E-stained sections of the hearts on day 14. Scale bars, 1 mm or 50 μm. (**B**) Myocarditis severity in heart sections (n = 6–7 per group). (**C**) Heart-to-body weight ratios (HW/BW) of the mice (n = 10 per group). Results are presented as the mean ± standard error of the mean. * *p* < 0.05 vs. WT.

As shown in Figure 3A, PD-L2^+/−^ mice, as well as PD-1^+/−^ mice, developed severe myocarditis with intense inflammatory infiltrates. In contrast, wild-type (WT) mice (F1 hybrids of WT C57BL/6J mice and BALB/c mice) developed weak myocarditis with lower myocarditis severity scores (Figure 3A,B). Heart-to-body weight ratios (HW/BW) in the PD-1^+/−^and PD-L2^+/−^ mice were significantly increased compared to WT mice (Figure 3C).

**Figure 4:**
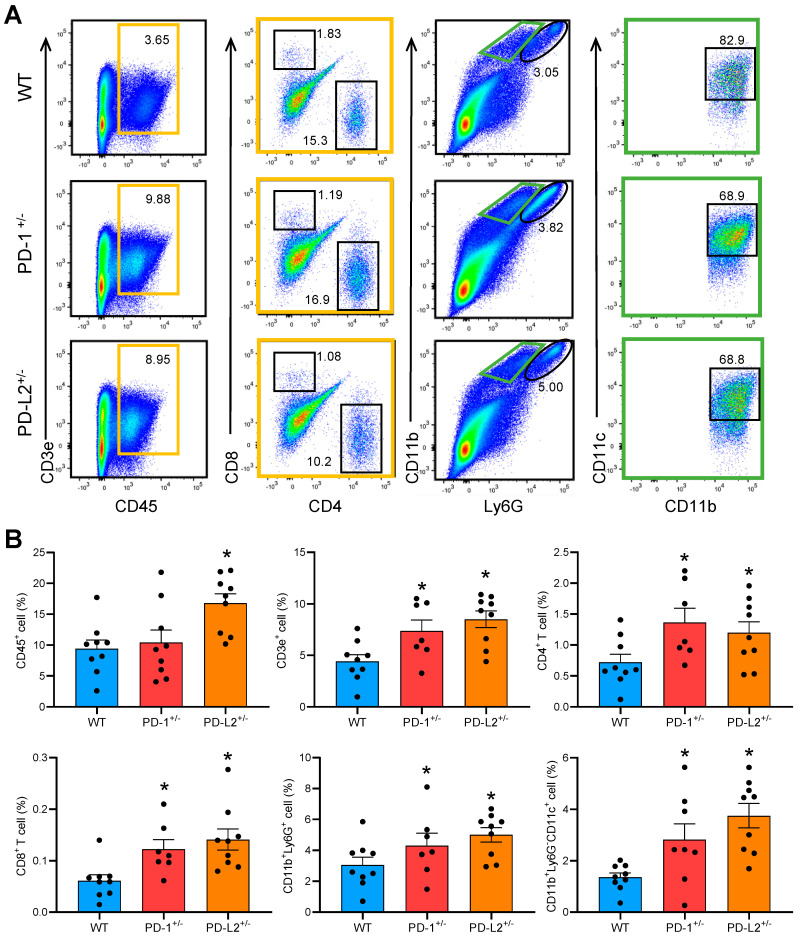
PD-L2 deficiency increases inflammatory cell infiltration in the experimental autoimmune myocarditis (EAM) heart. (**A**) Representative flow cytometric plots showing CD45^+^ leukocytes, CD3e^+^ lymphocytes, CD8^+^ T cells, CD4^+^ T cells, CD11b^+^Ly6G^+^ neutrophils, and CD11b^+^CD11c^+^ DCs from WT, PD-1^+/−^, and PD-L2^+/−^ hearts on day 14 after EAM induction. The orange squares indicate CD3e^+^CD45^+^ lymphocytes and the green squares indicate CD11b^+^Ly6G^dull^ cells. (**B**) Quantification of inflammatory cells in the hearts obtained from WT mice as well as PD-1^+/−^ and PD-L2^+/−^ mice with EAM. Results are presented as the mean ± SEM, n = 7–9 per group. * *p* < 0.05 vs. WT.

To analyze the impact of PD-L2 deficiency on inflammatory cellular infiltrate in the EAM hearts, flow cytometric analyses were performed on day 14 following immunization. The hearts of PD-L2^+/−^ mice with EAM showed significantly more inflammatory cell infiltration, such as an increased number of CD45^+^ leukocytes, CD3^+^ T cells, CD4^+^ T cells, CD8^+^ T cells, CD11b^+^Ly6G^+^ neutrophils, and CD11b^+^CD11c^+^ DCs, than in the hearts of WT mice with EAM (Figure 4A,B). PD-1^+/−^ mice with EAM also had a significantly greater number of infiltrating cells in the heart than WT mice with EAM (Figure 4A,B).

### 2.3. PD-L2 Deficiency Promotes Proinflammatory Cytokine Expression in the EAM Heart

**Figure 5:**
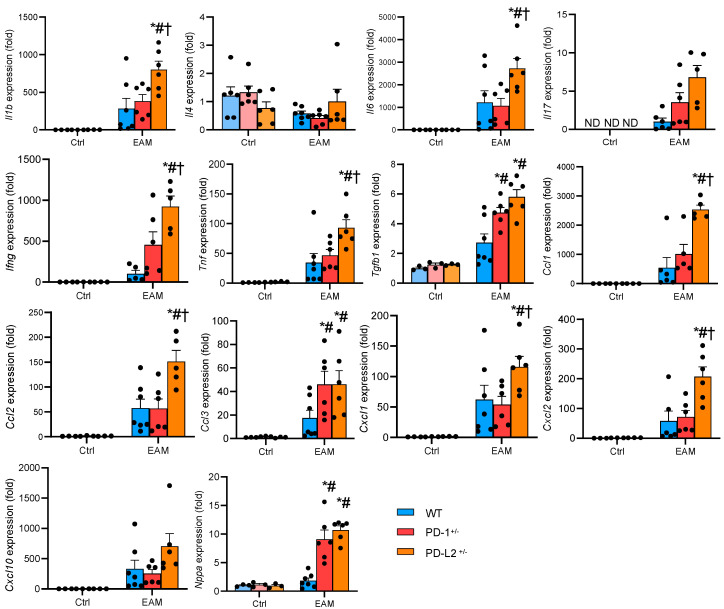
PD-L2 deficiency promotes proinflammatory cytokine expression in the experimental autoimmune myocarditis heart. mRNA expression of inflammatory markers in the hearts of wild-type (WT), PD-1^+/−^, and PD-L2^+/-^ mice at 14 days after immunization. Results are presented as the mean ± standard error of the mean. n = 3–6, * *p* < 0.05 vs. control, # *p* < 0.05 vs. WT, and † *p* < 0.05 vs. PD-1^+/−^.

To further evaluate the effects of PD-L2 deficiency on EAM, we examined cytokine and chemokine milieu in the heart. The heart homogenates from PD-L2 mice showed increased gene expression levels of cytokines such as interleukin (IL)-1β, IL-6, interferon (IFN)-γ, transforming growth factor (TGF)-β1, tumor necrosis factor (TNF)-α, chemokine (C-C motif) ligand (CCL)1–3, chemokine (C-X-C motif) ligand (CXCL) 1, 2, and 10, and atrial natriuretic peptide (ANP) (Figure 5). PD-1^+/−^ mice with EAM showed similar trends as PD-L2^+/−^ mice, but the expression levels were relatively weak (Figure 5).

### 2.4. PD-L2 Deficiency in Dendritic Cells Promotes CD4^+^ T Cell Proliferation

**Figure 6:**
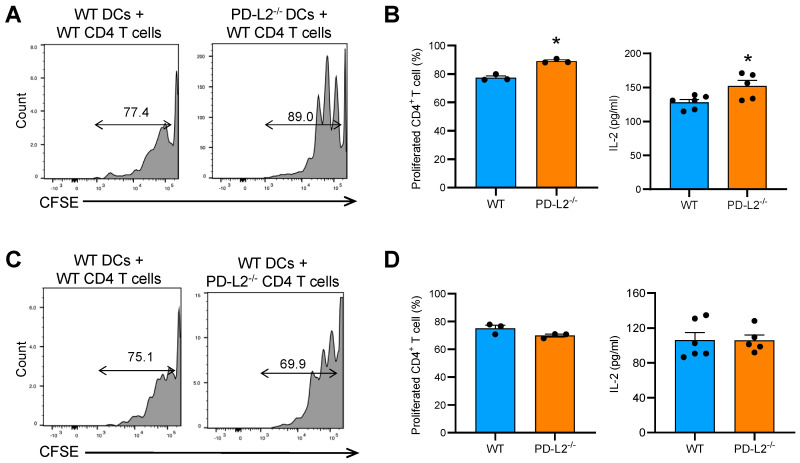
PD-L2 deficiency in dendritic cells (DCs) promoted CD4^+^ T cell proliferation. (**A**,**B**) Carboxyfluorescein succinimidyl ester (CFSE)-labeled WT CD4^+^ T cells were co-cultured with bone marrow-derived dendritic cells (BMDCs) generated from wild-type (WT) or PD-L2^−/−^ mice and stimulated with anti-CD3 and anti-CD28 for 72 h. Proliferation of CD4^+^ T cells was visualized by observing the incremental loss of CFSE fluorescence. Representative histograms (gated on CD4^+^ T cells) and the frequency of the proliferated CD4^+^ T cells among all CD4^+^ cells are shown. n = 3 per group. IL-2 concentrations in the culture supernatants were assessed by ELISA. n = 6 per group. Results are presented as the mean ± SEM. * *p* < 0.05 vs. WT. (**C**,**D**) CD4^+^ T cells were isolated from WT or PD-L2^−/−^ mice and labeled with CFSE. Then, they were co-cultured with WT BMDCs and stimulated with anti-CD3 and anti-CD28 for 72 h. Proliferation of CD4^+^ T cells was visualized by observing the incremental loss of CFSE fluorescence. Representative histograms and the frequency of the proliferated CD4^+^ T cells among all CD4^+^ cells are shown. n = 3 per group. IL-2 concentrations in the culture supernatants were assessed by enzyme-linked immunosorbent assay. n = 6 per group. Results are presented as the mean ± standard error of the mean.

EAM is a CD4^+^ T cell-mediated disease, and DCs are the major antigen-presenting cells and key players in the priming of appropriate CD4^+^ T cell responses [3]. To assess the effect of PD-L2 deficiency on CD4^+^ T cell function, we performed a co-culture experiment *in vitro.* First, we assessed whether PD-L2 deficiency in DCs affects CD4^+^ T cell proliferation. As shown in Figure 6A, WT CD4^+^ T cells co-cultured with bone marrow-derived DCs (BMDCs) from PD-L2^−/−^ mice showed significantly more proliferation than those co-cultured with WT BMDCs. In addition, CD4^+^ T cells co-cultured with PD-L2^−/−^ BMDCs produced significantly more IL-2 than WT BMDCs (Figure 6B). On the other hand, PD-L2^−/−^ CD4^+^ T cells co-cultured with WT BMDCs did not affect CD4^+^ T cell proliferation and IL-2 production (Figure 6C,D). These results suggest that PD-L2 expressed in DCs may inhibit T cell proliferation, thereby suppressing the development of autoimmune myocarditis.

## 3. Discussion

This is the first study to show that PD-L2 suppresses cardiac autoimmunity. Our study revealed a critical role of PD-L2 in controlling autoimmune heart disease. PD-L2 was sparsely detected in normal hearts but was upregulated under inflammatory conditions. PD-L2 deficiency accelerated myocardial inflammation in cardiac myosin peptide-induced EAM. PD-L2-deficient DCs enhanced CD4^+^ T cell proliferation in the presence of TCR and CD28 signaling. These findings suggest that PD-L2 in DCs may protect against cardiac antigen-reactive CD4^+^ T cell expansion and severe inflammation in autoimmune myocarditis.

PD-L1 and PD-L2 are the two known ligands of PD-1 and they exhibit different expression patterns. Unlike the widely expressed PD-L1 on the surface of many cell types, PD-L2 expression was originally reported to be restricted to activated DCs, macrophages, and bone marrow-derived mast cells [13]. In the present study, we found that PD-L2 was expressed on heart-infiltrating DCs and its expression increased with inflammation (Figure 2). More recently, PD-L2 expression has been detected in various cancers [14,15,16], resting peritoneal B1 cells [17], and activated T cells [18]. In our study, PD-L2 was transiently detected on the surface of heart-infiltrating CD4^+^ and CD8^+^ T cells during myocardial inflammation (Figure 2). The peak of PD-L2 expression on inflammatory cells was delayed compared to the peak of myocardial inflammation (Figure 1,2). PD-1/PD-Ls as immune checkpoint molecules downregulate T cell activity during immune responses [19]. Due to the nature of immune checkpoints, PD-L2 may play an important role in regulating immune responses to prevent autoimmune heart damage.

In the co-culture experiments, PD-L2 deficiency in DCs increased TCR- and CD28-mediated CD4^+^ T cell proliferation (Figure 6A), which was consistent with a published report that showed PD-L2-deficient splenic antigen-presenting cells (APCs) enhanced CD4^+^ T cell activation [20]. In the presence of anti-PD-1 antibody, CD4^+^ T cells exhibited similar proliferation when activated by WT or PD-L2-deficient APCs [20]. Together, these results indicate that PD-L2 may negatively regulate CD4^+^ T cell proliferation in a PD-1-dependent manner.

We observed that PD-L2 expression on CD4^+^ T cells was markedly upregulated on day 21 after EAM induction (Figure 2). However, PD-L2 deficiency in CD4^+^ T cells did not affect T cell proliferation (Figure 6B). In this study, we could not elucidate the role of PD-L2, expressed on CD4^+^ T cells, in the pathophysiology of EAM. Further investigation is needed to determine whether PD-L2 in CD4^+^ T cells is involved in the development and progression of autoimmune myocarditis.

Myocarditis is an inflammatory disease of the myocardium that is generally self-limited. However, in several cases, prolonged inflammation eventually results in cardiomyopathy, which is a potentially lethal disorder characterized by progressively impaired cardiac function [21,22]. Myocarditis can be triggered by many different environmental agents, including viral and bacterial infections, toxins, and drugs [23,24], and subsequent autoimmune response is thought to contribute to the disease progression to cardiomyopathy [25,26]. In this study, we clearly showed the protective roles of PD-L2 in cardiac autoimmune responses. Our findings suggest that the PD-L2 pathway might serve as a novel therapeutic target in the treatment of myocarditis.

## 4. Materials and Methods

### 4.1. Mice

PD-1^−/−^ mice were kindly provided by Dr. Honjo [4], and PD-L2^−/−^ mice were generated at the Laboratory Animal Resource Center, University of Tsukuba. BALB/c mice were purchased from CLEA, Japan. F1 hybrids of PD-1^−/−^or PD-L2^−/−^ mice on the C57BL/6J background and BALB/c mice were generated (PD-1^+/−^ and PD-L2^+/−^ mice, respectively). Male PD-1^+/−^ and PD-L2^+/−^ mice and WT littermates (6–8 weeks of age) were used in the in vivo experiments. All animal experiments were approved by the Institutional Animal Experiment Committee of the University of Tsukuba (approved number: 20037, approved date: 1 June 2020) and conformed to the NIH Guide for the Care and Use of Laboratory Animals.

### 4.2. EAM Induction

Mice were subcutaneously immunized with 100 μg of myosin heavy chain-α (MyHC-α) (MyHC-α_614–629_ [Ac-RSLKLMATLFSTYASADR-OH]; Toray Research Center) emulsified 1:1 in PBS/complete Freund’s adjuvant (1 mg/mL; H37Ra; Sigma–Aldrich) on days 0 and 7, as described previously [27,28,29]. On day 14, mice were euthanized, and their hearts were removed for further analysis.

### 4.3. Histopathological Examination

The hearts were fixed in 4% paraformaldehyde in PBS, embedded in paraffin wax, sectioned into 3-μm thick sections, and stained with hematoxylin and eosin for histological analysis. Myocarditis severity was scored in hematoxylin and eosin-stained sections using grades from 0–4: 0, no inflammation; 1, >25% of the heart section involved; 2, 25–50%; 3, >50–75%; and 4, >75%, as described previously [27]. The analysis was performed in a blinded manner.

### 4.4. Flow Cytometric Analyses

Heart inflammatory cells were isolated and processed as previously described [30,31]. For the flow cytometric analysis of the surface markers and cytoplasmic cytokines, the cells were stained directly using fluorochrome-conjugated mouse-specific antibodies and analyzed using the FACSVerse instrument (BD Biosciences, San Jose, CA, USA). The following monoclonal antibodies were used for flow cytometric analysis: Anti-CD279 (PD-1, TONBO Biosciences, San Diego, CA, USA), anti-CD274 (PD-L1, TONBO Biosciences, San Diego, CA, USA), anti-CD273 (PD-L2, Invitrogen, Waltham, MA, USA), anti-CD45 (TONBO Biosciences, San Diego, CA, USA), anti-CD4 (TONBO Biosciences, San Diego, CA, USA), anti-CD8 (eBioscience, San Diego, CA, USA), anti-CD11b (BioLegend, San Diego, CA, USA), anti-CD11c (TONBO Biosciences, San Diego, CA, USA), and anti-Ly6G (BD Pharmingen, San Jose, CA, USA).

### 4.5. RNA Extraction and Quantitative Real-Time Reverse Transcription Polymerase Chain Reaction

All hearts of mice, removed for performing reverse transcription polymerase chain reaction, were snap frozen and stored at −80 °C. Subsequently, the tissue was homogenized using the bead kit (MagNA Lyser Green Beads; Roche Diagnostics, Indianapolis, IN, USA) in accordance with the manufacturer’s instructions. Total RNA was extracted using the RNeasy Fibrous Tissue Mini Kit (Qiagen, Hilden, Germany) in accordance with the manufacturer’s instructions. cDNA was synthesized from 1 μg of total RNA per sample using the Omniscript RT kit (Qiagen, Hilden, Germany). A quantitative reverse transcription polymerase chain reaction was performed on the LightCycler 480 system (Roche Applied Science, Indianapolis, IN, USA) using a Universal Probe Library (Roche Applied Science, Indianapolis, IN, USA). Hypoxanthine-guanine phosphoribosyltransferase (HPRT) RNA was used as an internal control. Gene expression values were calculated using the 2^−ΔCt^ method.

### 4.6. T Cell Proliferation Assay

Naïve CD4^+^ T cells were isolated from splenocytes of mice using mouse CD4 (L3T4) MicroBeads (Miltenyi Biotec, Bergisch Gladbach, Germany). BMDCs were generated as previously described [32]. CD4^+^ T cells were labeled with 32 μL of 5 mM carboxyfluorescein succinimidyl ester (CFSE, Dojindo, Kumamoto, Japan) in 8 mL of PBS with 0.1% BSA for 10 min at 37 °C. The staining reaction was stopped with 1 mL of FCS (Lonza, Basel, Switzerland). CFSE-labeled 5 × 10^5^ CD4^+^ T cells were co-cultured with 10^5^ BMDCs and stimulated with 5 μg/mL of anti-CD3 and 3 μg/mL of anti-CD28 antibodies in 96-well round-bottom plates for 72 h. Proliferation of CD4^+^ T cells was visualized by observing the incremental loss of CFSE fluorescence using the FACSCalibur instrument (BD Biosciences, San Jose, CA, USA) and analyzed using FlowJo software (Tree Star, San Carlos, CA, USA).

### 4.7. ELISA

The concentrations of IL-2 in cell culture supernatants were measured using the DuoSet ELISA kits (R&D Systems, Minneapolis, MN, USA) in accordance with the manufacturer’s instructions.

### 4.8. Statistics

All data are expressed as the mean ± standard error of the mean (SEM). Normality was verified using the Shapiro–Wilk test. Statistical analyses were performed using the unpaired two-tailed *t*-test or Mann–Whitney U test for comparison between two groups. For multiple comparisons, one-way analysis of variance with the Newman–Keuls post hoc test or Kruskal–Wallis analysis with the post hoc Steel–Dwass or Steel test was performed. Results with *P* < 0.05 were considered as statistically significant. All statistical analyses were performed using JMP software (SAS Institute, Cary, NC, USA).

## 5. Conclusions

PD-L2 plays a pivotal role in suppressing cardiac autoimmunity. PD-L2 on DCs protects against autoreactive CD4+ T cell expansion and severe inflammation in mice with EAM.

## Data Availability

The data that support the findings of this study are available from the corresponding author (K.T.), upon reasonable request.

## Abbreviations

APC	antigen-presenting cell
BMDC	bone marrow-derived dendritic cell
CCL	chemokine (C-C motif) ligand
CFSE	carboxyfluorescein succinimidyl ester
CXCL	chemokine (C-X-C motif) ligand
DC	dendritic cell
EAM	experimental autoimmune myocarditis
H&E	hematoxylin and eosin
IFN-γ	interferon-γ
IL	interleukin
MyHC-α	myosin heavy chain-α
PD-1	programmed death 1
PD-L1	programmed death ligand 1
PD-L2	programmed death ligand 2
TCR	T cell receptor
TGF-β	transforming growth factor beta
TNF	tumor necrosis factor
WT	wild-type
